# Temporal Regulation of Rapamycin on Memory CTL Programming by IL-12

**DOI:** 10.1371/journal.pone.0025177

**Published:** 2011-09-23

**Authors:** Xiangdong Li, Karla Garcia, Zhifeng Sun, Zhengguo Xiao

**Affiliations:** Department of Animal and Avian Sciences, University of Maryland, College Park, Maryland, United States of America; University of Hyderabad, India

## Abstract

Mammalian target of rapamycin (mTOR) is a master regulator of cell growth. Recent reports have defined its important role in memory cytotoxic T lymphocyte (CTL) differentiation in infections and memory programming. We report that rapamycin regulated memory CTL programming by IL-12 to a similar level in a wide range of concentrations, and the enhanced memory CTLs by rapamycin were functional and provided similar protection against *Listeria Monocytogenes* challenge compared to the control. In addition, rapamycin-experienced CTLs went through substantially enhanced proliferation after transfer into recipients. Furthermore, the regulatory function of rapamycin on CD62L expression in memory CTLs was mainly contributed by the presence of rapamycin in the first 24-hr of stimulation *in vitro*, whereas the effective window of rapamycin on the size of memory CTLs was determined between 24 to 72 hrs. In conclusion, rapamycin regulates IL-12-driven programming of CTLs to a similar level in a wide range of concentrations, and regulates the phenotype and the size of memory CTLs in different temporal windows.

## Introduction

Induction of functional memory cytotoxic T lymphocytes (CTLs) is one of the main goals and challenges for vaccination [1], [2], [3], [4], [5], [6], [7], [8], [9], [10]. The molecular mechanisms for the generation of memory CTLs are just beginning to be understood. Antigen and co-stimulation have been shown to induce clonal expansion [11], [12], however, they are not sufficient to drive the differentiation and development of memory CTLs [5], [13], [14], in which case a third signal is needed. Inflammatory cytokines are induced in early infection [15], and are shown to provide a third signal to CTL fully activation [5], [13]. More importantly, memory CTL response is abolished in vaccinia virus and *Listeria monocytogenes* infections when receptors to both IL-12 and type I IFNs are lacking in CD8 T cells [16]. Interestingly, when naïve antigen-specific CD8 T cells were stimulated for 3 days with antigen, B7 and IL-12 in vitro, these cells developed into a functional memory population after transfer [16], indicating that programming of memory CD8 cells may take place during early activation.

mTOR is a serine/threonine protein kinase, which is highly conserved in evolution. It is a master regulator of cell growth and metabolism in response to environment factors, including cellular energy levels, insulin and other growth factors, amino acids, etc [17], [18], [19], which has been extensively investigated as a target in cancer therapy and transplant tolerance [19], [20], [21], [22]. Recently, mTOR has been shown to play a critical role in both innate and adaptive immune responses, notably in the regulation of dendritic cells, T and B cells [19], [23]. As an inhibitor of mTOR signaling, rapamycin has been commonly used in organ transplantation to prevent graft rejection, and in cancer therapy [20], [24], [25]. Surprisingly, administration of rapamycin to mice during LCMV infection promoted memory CD8 T cells through the inhibition of mTORC1 complex in CD8 T cells [26]. This indicates that memory CTL formation can be modulated by the regulation of cell metabolisms [27]. Pearce and colleagues reported that TRAF6 is required for memory CTL formation by affecting fatty acid oxidation (FAO) [28]. Administration of either antidiabetes drug metformin or rapamycin replaced this requirement, and restored memory CD8 T cells [28]. mTOR may regulate CD8 T cells by favoring anabolic metabolism in effectors during antigen and cytokine stimulation. Contrary to that, memory CD8 T cells can be enhanced by inhibition of mTOR by rapamycin or AMPK, which switches to catabolic from anabolic metabolism [27]. However, the way in which metabolic change regulates memory CTL differentiation remains unknown [27].

Recently, rapamycin was reported to program memory CTLs in the presence of IL-12 in vitro, by inhibition of CTL effector function but promoting memory potential, which increased memory CTL precursors and their survival [29]. However, how rapamycin regulated memory CTL differentiation, such as its optimal concentration and temporal requirements, have not been evaluated. By using the OT1 system, we found that rapamycin inhibited early activation of CTLs to a similar level in a wide range of concentrations, which equally enhances the generation of memory CTLs in the presence of IL-12. Moreover, temporal requirements are different for rapamycin in regulating the size and phenotype of memory CTLs.

## Materials and Methods

### Mice, cell lines, and reagents

OT-I mice (a gift from Dr. Mescher, University of Minnesota) having a transgenic TCR specific for H-2K^b^ and OVA_257–264_ [30] were crossed with Thy1-congenic B6.PL-Thy1a/Cy (Thy1.1) mice (Jackson ImmunoResearch Laboratories, Bar Harbor ME) and bred to homozygosity. The development of CD8 T cell in all strains appeared normal with respect to numbers, distribution and phenotype (data not shown). Mice were maintained under specific pathogen-free conditions at the University of Maryland, and these studies have been reviewed and approved by the Institutional Animal Care and Use Committee. C57BL/6 mice were purchased from the National Cancer Institute. All directly conjugated fluorescent antibodies were purchased from BD Biosciences, eBioscience or Biolegend. Rapamycin was purchased from EMD (Gibbstown, NJ). The dosage was 75 µg/kg/d [26] for rapamycin injection through i.p. in recipient B6 mice.

### Viruses and bacteria

Recombinant *Listeria monocytogenes* expressing full-length secreted ovalbumin (LM-OVA) was used for infection at 5×10^5^ i.v. for re-challenge, which was a gift from Dr. Jameson, University of Minnesota. Spleen cells from memory mice were analyzed by FACS for the percentage of OT1 cells in live cells, and bulk spleen cells containing 10^5^ memory OT1 cells were transferred into naïve B6 mice, which were then challenged by LM-OVA the next day at 5×10^5^ CFU/mouse i.v. Therefore, the comparison of memory protection was based on the same amount of memory CTLs among different groups. The spleen and liver were harvested three days after LM-OVA challenge, and LM-OVA was cultured using TSB plates for the comparison of protection as in our previous report [16].

### Naive T cell purification

This was performed in the same way we reported before [16]. Briefly, inguinal, axillary, brachial, cervical, and mesenteric lymph nodes (LNs) were harvested from WT OT-I mice, pooled, and disrupted to obtain a single cell suspension. CD8^+^CD44^lo^ cells were enriched by negative selection using MACS magnetic beads (Milteny Biotec). In brief, cells were coated with FITC-labeled antibodies specific for CD4, B220, I-A^b^, and CD44. Anti-FITC magnetic MicroBeads (Miltenyi Biotech) were then added and the suspension passed through separation columns attached to a MACS magnet. Cells that did not bind were collected, and were >95% CD8^+^ and <0.5% CD44^hi^. Purified naive OT-I cells were sorted to reach purity close to 100%.

### Real-time RT-PCR

RNA was isolated (Qiagen RNeasy mini kit) and used to synthesize cDNA (Qiagen QuantiTech Reverse Transcription kit). Quantitation was performed on a *MyiQ*™ *Single*-Color Real-Time PCR Detection System (Bio-Rad). Primers used were as follows: CD62L 5′ left primer, 5′-gctggagtgacacccttttc-3′; CD62L 3′ right primer, 5′-gttgggcaagttaaggagca-3′; GAPDH 5′ left primer, 5′-TGTCTCCTGCGACTTCAACAGC-3′; GAPDH 3′ right primer, 5′-TGTAGGCCATGAGGTCCACCAC-3′. Details of the real-time PCR conditions used are available upon request.

### Adoptive transfer and flow cytometric analysis

In vitro activated OT1 cells were adoptively transferred into normal C57BL/6NCr mice by i.v. (tail vein) injection at 10^6^ cells/mouse. Blood samples were drawn at indicated times, and the analysis of memory CTLs was based on the spleen and/or blood. Single cell suspensions were prepared, viable cell counts were performed (trypan blue) and the percent of OT-I cells in the sample was determined by flow cytometry. The adoptive transfer recipients were C57BL/6, and OT-I cells were identified as CD8^+^Thy1.1^+^ cells. Background for determining OT-I cell numbers was determined by identical staining of cells from normal C57BL/6 mice (no adoptive transfer). Analysis was done using a FACSCalibur™ flow cytometer and CELLQuest™ software (BD Biosciences) to determine the percent and total OT-I cells in the samples. Flowjo software (Tree Star Inc.) was used for analysis of the data.

### Intracellular cytokine staining after *in vitro* re-challenge

Spleen cells from adoptively transferred mice were incubated at 2×10^6^ cells/ml in RP-10 with 0.2 µM OVA_257–264_ peptide and 1 µl Brefeldin A (Biolegend) for 3.5 hrs at 37°C. Cells were then fixed in fixing buffer (Biolegend) for 15 min at 4°C, permeablized in Saponin-containing Perm/Wash buffer (Biolegend) for another 15 min at 4°C, and then stained with PE-conjugated antibody to IFNγ for 30 min at 4°C. Cells were then washed once with Perm/Wash buffer, and once with PBS containing 2% FBS.

### Intracellular staining for cell signaling molecules

Spleen cells from adoptively transferred mice were washed twice with cold PBS (4°C), and were then fixed with 2% paraformaldehyde for 20 min at 37°C. The cells were chilled on ice for 2 min, and washed twice with cold PBS. Permeablization was performed using 90% ice-cold methanol (stored at −20° C) on ice for 30 min. Permeablized cells were washed twice with cold PBS, and blocked for 10 min with 0.5% BSA-PBS at room temperature. Staining with primary and secondary antibodies was carried out for 30 min at 4°C. Cells were washed twice with 0.5% BSA-PBS after each staining.

### *In vitro* stimulation of naïve OT-I T cells

Naïve OT-I.PL T cells were purified as described above and stimulated for a certain time in vitro in flat-bottom microtiter wells coated with antigen (DimerX H-2Kb∶Ig fusion protein loaded with OVA_257–264_ peptide; BD Pharmingen) and recombinant B7-1/Fc chimeric protein (R&D Systems) as previously described [16]. 3×10^5^ cells in 1.5 ml Allos media were placed in each well and 2.5 U/ml IL-2 added to all wells (24-well plate). Where indicated, cultures were supplemented with 2 U/ml of murine rIL-12 (R&D Systems). Rapamycin stock (in DMSO) was diluted with corresponding culture medium as indicated. For the test of temporal windows, cells were washed three times after rapamycin treatment to remove residue rapamycin, which would then be put into fresh stimulation accordingly. Cells were harvested at the end of day 3, washed, resuspended at 3.33×10^6^ cells/ml in DPBS, and 10^6^ cells (300 µl) were transferred into C57BL/6 mice by tail vein injection. Cells that received IL-12 in vitro were termed 3 SI OT-I, and cells without IL-12 treatment were termed 2 SI OT-I. Transferred cells were identified by staining with anti-Thy 1.1 and anti-CD8 mAbs.

## Results

### Rapamycin delays CTL proliferation during early activation

**Figure 1:**
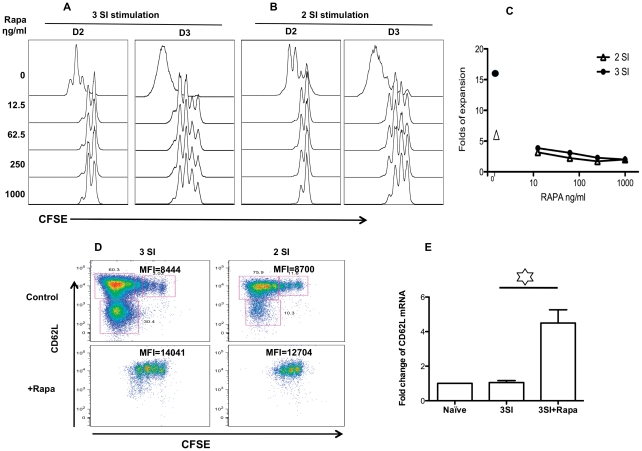
Rapamycin delays CTL proliferation during early activation. Sorted OT1 cells were labeled with CFSE, then stimulated with 2 SI (antigen+B7) or 3 SI (2 SI plus IL-12) in the presence of rapamycin at different concentrations. (A–B) Cell proliferation (CFSE dilution) was evaluated at day 2 and 3. (C) Fold expansion of CTLs 3 days after in vitro stimulation was calculated according to original input. (D) CD62L expression in CFSE labeled cells at day 3 after stimulation. Numbers beside or above each gate indicates the percentage of gated cells. MFI: Mean Fluorescence Intensity of the total population. These are representatives of at least three independent experiments with similar results. (E) Transcriptional regulation of CD62L by rapamycin. OT1 cells were stimulated for 3 days under 3 SI in the presence or absence of rapamycin, and then were harvested for real-time PCR examination. CD62L and housekeeping gene GAPDH were analyzed in triplicate in real-time RT-PCR assays. Relative mRNA amounts were normalized with respect to expression levels in Naïve OT1 control (fold change = 1). The results are expressed as mean+SEM of three independent experiments.

mTOR is involved in immune regulation by influencing DC's function, especially the production of signal 3, including both IL-12 and type I IFN [19], [23], [31]. Consistent with its main function as a regulator for cell growth and proliferation [18], mTOR is a mediator in CD4 cell cycle progression driven by integrated signals from both TCR and CD28 through the PI3K-AKT-mTOR pathway [19], [32]. Recently, inhibition of mTOR by rapamycin in CD8 T cells was shown to enhance memory T cell differentiation [26], [28]. To investigate the nature of rapamycin regulation on CTL proliferation during early activation, sorted naïve OT1 cells were labeled with *carboxyfluorescein diacetate succinimidyl ester* (CFSE), and stimulated with plate bound antigen and B7 (2-SI/2 signals), or 2-SI plus IL-12 (3-SI/3 signals). Rapamycin was diluted to span a wide range, and cell division was examined at day 2 or 3. Cells did not start to divide until day 2 after stimulation (day 1 data not shown). In 3-SI stimulation, cells division was delayed by at least two rounds by rapamycin, and there was not much difference among concentrations of rapamycin in a range of 12.5 to 1000 ng/ml (Fig. 1A), consistent with previous notion that in the presence of optimal IL-2, rapamycin can only delay but not prevent division in CD4 T cells [32], [33]. This may reflect the fact that rapamycin cannot completely inhibit the function of mTOR [34].

Rapamycin suppressed the cell proliferation of CTLs under 2-SI stimulation similarly to 3-SI stimulation (Fig.1B), indicating that rapamycin inhibits CD8 cell cycle progression independently of third signal cytokine. However, the inhibition of rapamycin on 3-SI stimulated cells was stronger than on 2-SI, which was indicated by cell yield after three days of stimulation (Fig. 1C). This may suggest that rapamycin causes stronger inhibition on the proliferation of faster dividing cells, as IL-12 enhances CTL clonal expansion in addition to promote effector functions [11], [13]. Rapamycin enhanced the expression of CD62L in 3 signal stimulations (Fig. 1D), which is consistent with one recent report [29]. Similar up-regulation of CD62L was observed in 2-SI stimulation (Fig.1D), and down-regulation of CD62L in both 2-SI and 3-SI conditions happened only in cells with more than 3 divisions in both cases (Fig.1D). Furthermore, the enhanced CD62L expression by rapamycin was related to increased CD62L transcription. The mRNA of CD62L was increased by about 4-fold when rapamycin was present in 3 signal stimulation for 3 days (Fig. 1E), indicating that the regulation of CD62L by rapamycin may be on transcription level.

### Rapamycin suppresses effector function of CTLs

**Figure 2:**
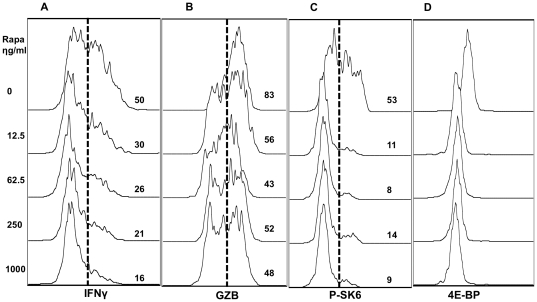
Rapamycin suppresses effector function of CTLs in wide range of concentration. Sorted OT1 cells were stimulated with antigen+B7+IL-12 in the presence of rapamycin at different concentrations. Programmed CTLs were harvested at 48 hrs. (A) The expression of IFNγ. (B) The expression of granzyme B (GZB). (C) The expression of P-SK6. (D) The expression of 4E-BP. These are representatives of three independent experiments with similar results.

IFNγ production is a hallmark of type I effector function, which is not affected by rapamycin treatment for 72 hrs [29]. We found that rapamycin had inhibitory effects on IFNγ production at 48 hrs after 3-SI stimulation in a dose-dependent manner (Fig. 2A), which was greatly reduced at 12.5 ng/ml, with maximal decrease at the highest rapamycin concentration (Fig. 2A). This suggests that mTOR is required in the initiation or early induction of IFNγ. Moreover, although IFNγ was greatly inhibited by rapamycin at 48 hrs (Fig. 2A), there was no difference at 72 hrs (data not shown), suggesting that mTOR may not be required for the maintenance of IFNγ production. Alternatively, other factor(s) may compensate this mTOR inhibition.

Granzyme B (GZB) is a molecule directly related to the killing function of CTLs [13]. Consistent with the recent report by Rao and colleagues [29], GZB production was suppressed by rapamycin at 48 hrs (Fig. 2B) and 72 hrs (data not shown). To confirm that the outcomes were from mTOR function, p70SK6 and 4E-BP1, 2 direct downstream targets of mTOR, were examined for their expression. Inhibition of mTOR by rapamycin greatly suppressed the expression of these two molecules at 48 hrs after stimulation, in a similar pattern by a wide range of concentrations, supporting that mTOR signal pathway is inhibited by rapamycin (Fig. 2C and D).

### Rapamycin experienced CTLs increase proliferation and maintain higher CD62L expression during memory differentiation

**Figure 3:**
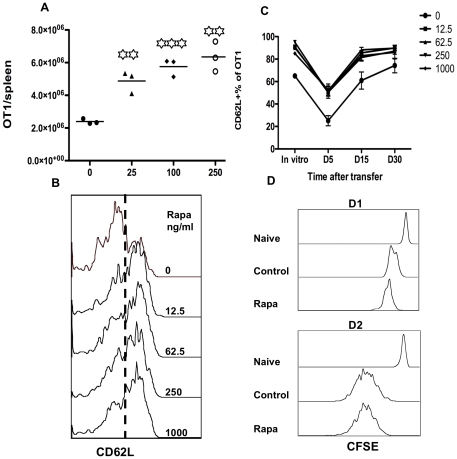
Rapamycin experienced CTLs have increased proliferation and maintain higher CD62L expression during memory differentiation. Experimental setting was the same as in Figure 2. Activated CTLs were harvested at day 3 after stimulation in vitro, and were transferred into naïve B6 mice at 10^6^/mouse. Blood samples were drawn at day 5, 15 and 30 after transfer. (A) Numbers of OT1 cells in spleen 5 days after transfer. Every group was compared to control group (rapamycin 0). (B) Representative CD62L expression in OT1 cells in blood at day 5 after transfer. (C) Comparison of CD62L expression in OT1 cells during memory differentiation. “In vitro” represents samples harvested at day 3 after stimulation in vitro. (D) In a separated experiment, rapamycin+3-SI programmed and control CTLs under 3-SI stimulation were harvested at 72 hrs, and labeled with CFSE before transferred into B6 mice. CFSE dilution was examined in OT1 cells from spleens at days 1 and 2 after transfer. Experiments are representatives of at least three independent experiments with similar results (A–C) or one experiment (D). Each value represents the mean plus the SD of 5 mice per group in C.

The differentiation of memory CTLs takes a long time before stable memory is established. To track this memory generation from CTLs regulated by different concentrations of rapamycin, we examined the kinetics of OT1 cells at different time points after transfer. In line with a previous report [16], CTLs activated by IL-12 experienced a significant expansion at day 5 after transfer in spleen (Fig. 3A), indicating that rapamycin enhanced the expansion potential of IL-12 conditioned CTLs. This difference was similarly reflected in blood of recipient mice throughout the memory differentiation (data not shown). This observation suggests that the regulatory function of rapamycin on IL-12 conditioned CTLs is likely contributed by an enhanced expansion potential and subsequent memory differentiation, but we cannot exclude the possibility of enhancing memory precursors and their survival [29].

There was a subtle difference (about 20%) in CD62L expression in spleen cells between rapamycin treated and control 5 days after transfer (Fig. 3B). This difference maintained throughout the whole experiment (Fig. 3C). Interestingly, activated OT1 quickly down-regulated CD62L at day 5 after transfer (Fig. 3B), and then up-regulated it at day 15, and reached a high level at day 30 (Fig. 3C). This rapamycin-induced CD62L up regulation has been shown to cause enhanced migration of CTLs to lymphoid tissues compared to controls [29], consistent with the function of CD62L as a homing molecule for secondary lymphoid tissues. One of the major functions of rapamycin is inhibiting cell proliferation, which was shown in vitro stimulation (Fig. 1). It was possible that rapamycin-treated CTLs would go through division slower than no rapamycin control. To address this possibility, CTLs programmed by IL-12 in the presence or absence of rapamycin for 3 days were labeled with CFSE, and were then transferred into recipient B6 mice at 10^6^/mouse. At days 1 and 2 after transfer, OT1 cells in spleen were examined for proliferation. These stimulated cells started to proliferate 24 hrs after transfer, and rapamycin-conditioned CTLs were about one round faster than controls (Fig. 3D), indicating that the difference of memory generation could be due to difference in the proliferating speed during memory differentiation. Thus, the enhanced expansion by rapamycin (Fig. 3A) could be related to increased proliferation (Fig. 3D) in addition to enhanced survival as suggested by Rao et al [29].

### Rapamycin regulates memory CTL programming in the presence of IL-12 in a wide range of concentrations

**Figure 4:**
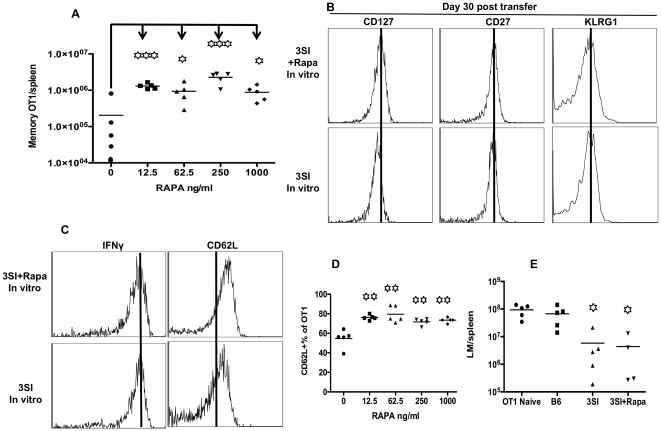
Rapamycin programs memory CTLs in the presence of IL-12 in early activation. Sorted OT1 cells were stimulated with antigen+B7+IL-12 in the presence of rapamycin at different concentrations. Programmed CTLs were harvested at 72 hrs, and transfer into naïve B 6 mice at 10^6^/mouse through tail injection. (A) Memory OT1 cells in spleen at day 30 after transfer. (B–D) Comparison of molecular expression in memory CTLs programmed by rapamycin (at 250 ng/ml) with control (no rapamycin). (E) Spleen cells containing 10^5^ memory OT1 from (B) were transferred into naïve B6 mice, which were challenged the next day with 5×10^5^ CFU LM-OVA through i.v. Bacterium counts were examined 3 days after LM-OVA challenge in spleen. Every group was compared to control group (OT1 naïve–10^5^ naive OT1 transfer group). These are representatives of two independent experiments with similar results. Every group was compared to control group (rapamycin 0) in A and D. Asterisks indicate statistical significance. *, P<0.05; **, P<0.01; ***, P<0.001. ns: not significant.

Inhibition of mTOR by rapamycin in the presence of IL-12 has been shown to enhance memory CTL programming by increasing the number of precursors and their survival [29]. We sought to understand how the strength of mTOR inhibition would affect CTL memory differentiation after programming in vitro. Indeed, rapamycin significantly enhanced memory programming by IL-12 with no much difference in a wide range of concentrations — 5 to 10-fold increase compared to no rapamycin control (Fig. 4A). This increased memory CTLs could not be contributed only by the survival of precursors, because the initial transfer number of in vitro programmed CTLs was only one million, and parking rate for normal transfer is about 10% [35], [36]. Therefore, there is a significant expansion based on the final memory size (Fig. 4A). Actually we have shown that CTLs programmed by IL-12 in vitro undergo vigorous expansion after transfer [16]. Interestingly, the memory CTLs in all groups demonstrated similar phenotypes in the expression of CD27, KLRG1 and IFNγ (Fig. 4B and C). There was a consistent marginal up regulation of CD127 (IL-7Rα) in rapamycin-induced CTLs, which could be related to improved survival based on the function of IL-7. Expression of CD62L was slightly but significantly up regulated by about 20% in rapamycin-treated memory CTLs at all concentrations (Fig. 4C and D). Equal numbers of memory CTLs were transferred into recipient naïve B6 mice, and the protection of these differentiated memory CTLs were examined by challenging with LM-OVA. Rapamycin treated memory CTLs demonstrated equal protection to LM-OVA in spleen compared to no rapamycin control (Fig. 4E), indicating that these rapamycin-programmed memory CTLs are equally functional compared to controls. Similar protection was found in liver (data not shown).

### Rapamycin regulates the size of memory CTLs and CD62L expression in different temporal windows

**Figure 5:**
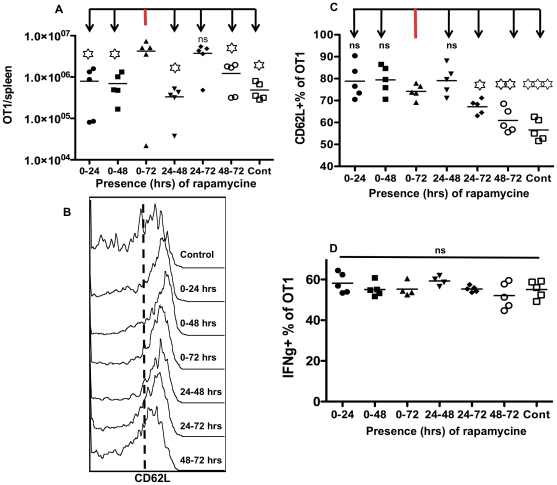
Rapamycin regulates memory CTLs and CD62L expression in different time windows. Sorted OT1 cells were stimulated with antigen+B7+IL-12 in the presence of rapamycin at 250 ng/ml for different windows. Programmed CTLs were harvested at 72 hrs, and transfer into naïve B6 mice at 10^6^/mouse. Memory OT1 cells were examined in spleen at day 30 after transfer for analysis of memory size (A), expression of CD62L (B–C) and production of IFNγ (D). Every group was compared to control group (0–72) in A and C.

Rapamycin enhanced IL-12-driven memory programming by 5 to 10-fold (Fig. 4A), but it might not be required to be present for the whole 3-day culture. We were interested in the effective temporal window for this regulatory function of rapamycin. Sorted naïve OT1 cells were stimulated with antigen, B7 and IL-12 for three days, in which rapamycin was present for different periods of time. Activated CTLs were transferred into naïve B6 mice, and memory CTLs were analyzed at 30 days after transfer. The memory size was the same level when rapamycin was present for the whole 72 or 24–72 hrs. The rest windows, either one day or two days, all generated significantly lower memory CTLs, at similar level to no rapamycin control (Fig. 5A) This indicates that the regulatory effects of rapamycin on memory size is mainly contributed by its function after 24 hrs.

However, rapamycin treatment regulated the expression of CD62L in a different window. CD62L expression was up regulated to a similar level when rapamycin was present at the first or second day in vitro (Fig. 5B and C). In contrast, the presence of rapamycin at the last 24 hrs seems to have either minimum or inhibitory effects on CD62L expression, indicating that effective window differs in the regulation of memory size and CD62L expression (Fig. 5A–C). Interestingly, memory CTLs showed similar capacity in IFNγ production in all the temporal windows of rapamycin (Fig. 5D), and expression of CD127 and KLRG1 was similar in all groups (data not shown). These data suggest that rapamycin regulates different aspects of memory CTL programming in different temporal windows.

### Injection of rapamycin in recipients can enhance memory CTL programming by IL-12 after transfer

**Figure 6:**
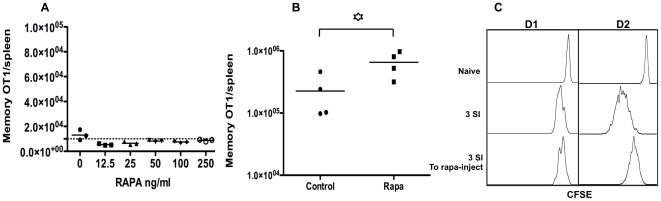
Injection of rapamycin in recipients can enhance memory CTL programmed by IL-12 after transfer. (A) Sorted OT1 cells were stimulated with antigen+B7+rapamycin (at different concentration) in the absence of IL-12. Stimulated CTLs were harvested at 72 hrs, and transfer into naïve B6 mice at 10^6^/mouse. Memory OT1 cells in spleen were examined at day 30 after transfer of CTLs. These are representatives of three independent experiments with similar results. (B) Sorted OT1 cells were stimulated with antigen+B7+IL-12, and were transferred into recipient B6 mice after 3 days. Half of the recipients received daily injection of rapamycin at 75 ug/kg from −1 to day 10 after transfer [26]. Memory CTLs in spleen were examined at day 30 after transfer. These are representatives of two independent experiments with similar results. (C) Sorted OT1 cells were stimulated with antigen+B7+IL-12 for 3 days, and then were labeled with CFSE before being transferred into recipient B6 mice at 10^6^/mouse. Half recipients received daily injection of rapamycin at 75 ug/kg from −1 to day 2 after transfer. CFSE dilution was examined at days 1 and 2 after transfer. Control: CFSE-labeled naïve OT1 cells. These are representatives of five animals in each treatment.

Although only antigen and B7 are unable to program memory CTLs [16], it was not known if rapamycin could replace the function of third signal cytokines, in other words, if rapamycin could program memory CTLs in the absence of a third signal. Indeed, CTLs programmed by 2-SI plus rapamycin failed to form memory CTLs, and had a similar level as 2-SI control (Fig. 6A), which confirmed that rapamycin cannot replace third signal to memory CTL programming. This furthers the notion that inflammatory cytokines are unique in providing a third signal to full activation of CTL and memory differentiation [5], [37]. However, rapamycin can influence many immune cells such as DCs [19], [23], which may affect memory CTL differentiation. We sought to find if administration of rapamycin in vivo could enhance memory CTLs programmed by IL-12 in vitro. Activated CTLs stimulated by 3-SI were transferred into recipient mice, and half of them received rapamycin treatment. Injection of rapamycin caused enhanced memory (Fig. 6B), suggesting that inhibition of mTOR during memory differentiation can also program memory CTLs. This enhancement was not as great as in vitro programming (Fig. 4A), which may be due to the availability of small amount of IL-12 in vivo after transfer. This low dosage of rapamycin is effective in mTOR regulation, because it could enhance memory CTLs by 3 to 4-fold in vaccinia virus infection (unpublished data). In addition, the low dosage of rapamycin injection caused obvious delay in CTL proliferation 24 hours after transfer, and the delay was still visible at day 2, even though no clear individual division was observed due to the limit of CFSE dilution (Fig. 6C). So, this memory enhancement was likely related to increased survival by rapamycin after transfer.

## Discussion

Functional effects of chemicals are normally closely related to their concentration. As an inhibitor for mTOR, rapamycin is usually used at around 25 ng/ml for CD8 T cell stimulation [29], [38]. We sought to find if stronger inhibition on mTOR function could be achieved with increased concentrations of rapamycin. Surprisingly, no much difference was observed in both in vitro and in vivo with a 80-fold range of rapamycin from 12.5 to 1000 ng/ml, suggesting that rapamycin functions at a similar level with this wide range of concentrations. This is supported by four lines of evidence. The first evidence is proliferation. All of the doses had similar inhibition on CTL division (Fig. 1A and B). Second, CD25, GZB and CD62L were similarly regulated by rapamycin at different concentrations (Fig. 2B and data not shown). Third, the downstream molecules of mTOR pathway, p70SK6 and 4E-BP1 [39], were suppressed to a similar level. Fourth and foremost, the rapamycin generated a comparable size of memory CTLs at all concentrations (Fig. 4A). All of the data point to one single conclusion that the wide range of rapamycin exerts similar biological function on CTL activation and programming.

It has been shown that rapamycin cannot completely stop the progress of cell cycle in CD4 T cells in the presence of optimal IL-2 [32], [33], which may be the same case for CD8 T cells. However, it is surprising that rapamycin demonstrated almost identical regulatory functions within a wide range of concentrations on the expression of many functional molecules, especially memory programming in this report.

Regulation of rapamycin on memory programming by IL-12 has been suggested to work through inhibition of CTL effector functions, such as transcriptional factor T-bet, and promoting eomes expression [29]. In this report, we also found that activation of IL-12 conditioned CTLs were inhibited by rapamycin, indicated by reduced production of Granzyme B (Fig. 2B), which is consistent with a previous report [29]. In addition, there was no difference in the expression of CD44 and CD127 by rapamycin treatment (data not shown). IFNγ was inhibited at 48 hrs, but not 72 hrs, indicating that mTOR is important for IFNγ initial induction, but not for the later stage, which is in line with a previous report [29]. The reduced recruitment of STAT4 and STAT3 by rapamycin [40] may be the mechanism for reduced IFNγ induction at 48 hrs in IL-12 conditioned CTLs (Fig. 2A). However, we could not rule out the possibility that the reduced IFNγ production may also be contributed by the general inhibition on protein translation regulated by rapamycin. In addition, during the continuous presence of rapamycin, IFNγ production was back to normal at 72 hrs (data not shown), suggesting that the regulation of IFNγ induction may use different mechanisms (factors) at different stages during early activation.

Although memory programming of rapamycin is dependent of IL-12 (Fig. 6A), the inhibitory function of rapamycin on CTL proliferation is independent of IL-12. It suppressed proliferation of 2-SI and 3-SI conditioned CTLs at a similar level (Fig. 1A–C). Clear inhibition of CTL proliferation by rapamycin was observed at 48 and 72 hrs (Fig. 1A and B), but not 24 hrs (data not shown), which overlaps with the temporal window (24 to 72 hrs) for the regulation of rapamycin on memory size (Fig. 5A). This may suggest that memory regulation by rapamycin is accompanying CTL dividing. We found that slow dividing CTLs programmed by IL-12 generated better memory than fast dividing ones after transferred into recipients (unpublished data). This may be related to the competition for limited resources for survival and growth, as fast dividers demand more. So the inhibition of rapamycin on CTL proliferation could be one of the mechanisms for enhanced memory programming by IL-12. However, slowing down proliferation by rapamycin could not generate memory under 2-SI stimulation (Fig. 6A), supporting the notion that third signal cytokine is indispensable for memory programming.

Although significantly enhanced memory CTLs were generated when rapamycin was present for 3 days in IL-12 stimulation, it might not be required for the whole 3-day incubation. Indeed, treatment of rapamycin for the last 48 hrs (24 to 72 hrs) induced comparable memory CTLs to controls (Fig. 5A). The rest of windows, either 24 or 48 hrs, led to significantly reduced memory CTLs compared to 3-day rapamycin control (Fig. 5A). Interestingly, Shrikant group found that delayed addition of rapamycin by 12 hrs at the beginning did not affect its regulation on type I effect function [29], which agrees with the delayed effective window for rapamycin regulation on memory size (Fig. 5A). However, a different temporal window is responsible for the regulation of CD62L expression, the most critical marker for central memory phenotype [4], [14]. CD62L expression was up regulated by the presence of rapamycin for the first 24 hrs, even though the number of memory CTLs was low (Fig. 5B and C). This suggests that short experience of rapamycin may help to generate central memory CTLs. In contrast, the presence of rapamycin in the last 24 hrs was detrimental for CD62L expression. Therefore, although the number of memory CTLs programmed by rapamycin in vitro was similar between 0–72 and 24–72 hrs, the CD62L expression at the later time was significantly lower, suggesting that the whole 3 days period may be required for full differentiation of functional memory CTLs with a central memory phenotype. Interestingly, the enhanced CD62L expression by rapamycin seems to be at the transcription level. mRNA of CD62L was increased by about 4-fold by rapamycin compared to control (Fig. 1E), consistent with a recent report by Sinclair et al [39], [41]. CD62L transcription is regulated by transcription factor KLF2, and KLF2 is suppressed by PI3K-mTOR pathway [39], [41]. Thus, inhibition of mTOR by rapamycin diminished this suppression of KLF2, which led to upregulation of CD62L transcription (Fig. 1E).

In vitro programmed CTLs undergo dramatic proliferation shortly after transfer, as shown in our previous report [16], and this proliferation can only happen in IL-12 conditioned CTLs. Similar results were observed in this report, and rapamycin treatment dramatically enhanced this proliferation and expansion (Fig. 3A). Interestingly, the effects of rapamycin on memory CTLs were more dramatic compared to the effects reported by Rao et al [29]. We obtained more memory OT1 cells than they did (about 5 fold more), and only one million cells were used for each transfer whereas 2 millions in their report [29]. It is not clear what caused this discrepancy, which could be related to differences in experimental systems. Nevertheless, we observed similar trend for rapamycin regulation on memory CTL programming by IL-12, which is related to enhanced expansion potential of CTLs after transfer. However, we could not exclude the possibility of an increase in memory CTL precursor and their subsequent survival, as proposed by Rao and colleagues [29].

Rapamycin cannot replace IL-12 in providing a third signal for CTL activation — it has to work with a third signal, but not as a third signal (Fig. 6A). Late supply of rapamycin led to enhanced memory (Fig. 6B), suggesting that interaction of rapamycin with small amount of IL-12 or other third signal in vivo could enhance memory, such as type I IFN. Type I IFN is the major cytokine for CTL expansion and subsequent memory differentiation in LCMV infection [42]. Interestingly, administration of rapamycin into LCMV infected mice results in increased memory CTLs [26]. We hypothesize that the combination of rapamycin and inflammatory cytokine such as IL-12 could serve as adjuvant for vaccination targeting protective memory CTLs. This adjuvant will be completely different from traditional adjuvants in that this is based on interactions between different pathways, rather than individual pathway initiated by single ligand-receptor recognition.
